# A Spectroscopic Study on PtCl_4_^(2−)^ Binding to Rabbit Skeletal Muscle G-Actin

**DOI:** 10.1155/MBD.1995.127a

**Published:** 1995

**Authors:** Juan Zou, Hong-Ye Sun, Kui Wang

**Affiliations:** 1 State Key Laboratory of Coordination Chemistry Coordination Chemistry Institute Nanjing University Nanjing 210008 China; 2 Inorganic Chemistry Department School of Pharmaceutical Sciences Beijing Medical University Beijing 100083 China

## Abstract

It was found that the binding of PtCl_4_^2−^ to G-actin and the consequent conformational changes are different with those for hard acids. It is a two-step process depending on molar ratio PtCl_4_^2−^/actin (R). In the first step, R less than 25, the PtCl_4_^2−^ ions are bound to sulfur-containing groups preferentially. These high-affinity sites determined by Scatchard approach are characterized by n_1_ = 30 with average binding constant K_1_=1.0×10^7^M^-1^. The conformational changes are significant as characterized by N-(1-pyrenyl) maleimide(NPM) labeled fluorescence, intrinsic fluorescence and CD spectra. EPR spectroscopy of maleimide spin labeled(MSL) actin demonstrated that even PtCl_4_^2−^binding is limited to a very small fraction of high-affinity sites(R<1), it can bring about a pronounced change of conformation. In the range of R=25-40, high-affinity sites accessible are saturated. In the second step(R>40) , deep-buried binding sites turn out to be accessible as a result of the accumulated conformational changes. These new binding sites are estimated to be n_2_=26 with average binding constant K_2_=2.1×10^6^M^-1^. Although in this step the quenching of intrinsic fluorescence goes on and the NPM-labled thiols moves to more hydrophilic environment, no change in α-helix content was found. These results suggested that with increasing in PtCl_4_^(2−)^ binding, the G-actin turns to an open and loose structure in a discontinuous mode.


### A SPECTROSCOPIC STUDY ON $PtCl_4^{(2-)}$ BINDING TO RABBIT SKELETAL MUSCLE G-ACTIN

Juan Zou[1] , Hong-Ye Sun[2] and Kui Wang*[2]

[1] State Key Laboratory of Coordination Chemistry, CoordinationChemistry Institute, Nanjing University, Nanjing 210008, China
[2] Inorganic Chemistry Department, School of Pharmaceutical Sciences, Beijing Medical University, Beijing 100083, China

### Abstract

It was found that the binding of $PtCl_4^{2-}$ to G-actin and the consequent conformational changes are different with those for hard acids. It is a two-step process depending on molar ratio $PtCl_4^{2-}$/actin (R). In the first step, R less than 25, the $PtCl_4^{2-}$ ions are bound to sulfur-containing groups preferentially. These high-affinity sites determined by Scatchard approach are characterized by $n_1$=30 with average binding constant $k_1=1.0x10^7M^{-1}$. The conformational changes are significant as characterized by N-(1-pyrenyl) maleimide(NPM) labeled fluorescence, intrinsic fluorescence and CD spectra. EPR spectroscopy of maleimide spin labeled(MSL) actin demonstrated that even $PtCl_4^{2-}$binding is limited to a very small fraction of high-affinity sites(R<l), it can bring about a pronounced change of conformation. In the range of R=25-40, high-affinity sites accessible are saturated. In the second step(R>40), deep-buried binding sites turn out to be accessible as a result of the accumulated conformational changes. These new binding sites are estimated to be $n_2$=26 with average binding constant $k_2=2.1x10^6M^{-1}$. Although in this step the quenching of intrinsic fluorescence goes on and the NPM-labled thiols moves to more hydrophilic environment, no change in $\alpha$-helix content was found. These results suggested that with increasing in $PtCl_4^{2-}$ binding, the G-actin turns to an open and loose structure in a discontinuous mode.

### Introduction

DNA has been generally accepted to be the main target of anticancer complex, cisplatin[1,2]. However, evidences accumulated in recent years support that there are targets other than DNA in the cell. Among them, cytoskeletal system has been found susceptible to the attacking platinum complexes. Kopf-Maier et al[3] and Su[4] reported separately the disorganization of microtubule, medium microfilament as well as microfilament network caused by cisplatin. Aggarwal also reported the effect of cisplatin on cytoplasmic actin filaments from qualitative histological and ultrastructural observations[5,6].

In our previous work[16], we found the platinum binding to F-actin causes changes in conformation and association, but the mechanism of these effects is still unclear. In the present work, the nature of binding of $PtCl_4^{2-}$ to monomeric actin(G-actin) and the subsequent conformational changes were studied by spectroscopic methods.

### Material and Methods

### Chemicals

ATP(disodium salt), sodium azide, NPM and N-(1-oxyl-2,2,6,6-tetra-methyl-4-piperidinyl) maleimide were purchased from Sigma Chemical Co. and used without further purification. All other reagents were of analytical grade and all solutions were prepared with deionized water. A 1.02mM $K_2PtCl_4$ solution was prepared and calibrated by means of AAS method. Buffer A was composed of 2mM Tris, 0.2mM $CaCl_2$, 0.005% $NaN_3$ and pH8.0.

### Purification of G-actin

G-actin was extracted from rabbit muscle acetone powder and purified according to the method of Pardee and Spudich[7]. Free nucleotide and 2-mercaptoethanol were removed using a Sephadex G-25 column (preequilibrated with buffer A). G-actin concentration was determined according to the absorbance at 290nm with the absorption coefficient $A_{290}^{1mg/ml}$=0.63[8]. The level of impurities in the actin is less than 0.5% as determined by SDS-polyacrylamide gel electrophoresis[9].

### Fluorescence Studies

All the steady fluorescence measurements were conducted with a Shimadzu RF-540 spectrophotofluorimeter at room temperature. Slits for both excitation and emission were set at 10nm.

### Intrinsic Fluorescence

Samples were prepared by incubating aliquots of G-actin solution (0.4μM) with various amounts of $K_2PtCl_4$ for approximately 10h at 4°C. The $K_2PtCl_4$ concentration is varied in the range of 0-40μM. The intrinsic fluorescence was measured with $\lambda_{ex}$=286nm and $\lambda_{em}$=340nm.

The Reaction of $PtCl_4^{2-}$-actin Complexes with NPM

$PtCl_4^{2-}$-actin solution was prepared by incubating G-actin (0.4μM) with various volume of $K_2PtCl_4$ solution. After standing at 4°C for 10h, 7μl 3mM NPM solution was added to each of the solutions, and set overnight at 4°C, then fluorescence measurements were conducted. After the samples were dialyzed against the buffer A to remove the free NPM, the bound NPM was estimated[10] from the absorbance at 345nm (for pyrene chromophore) with the molar absorptivity as $4x10^4cm^{-1}M^{-1}$.

The Reaction of NPM Labeled G-actin with $PtCl_4^{2-}$

G-actin was labeled with NPM by Kawasaki's method[11], with molar ratio of NPM to actin 1.02. After standing 2-4h at 4°C and dialyzing against the buffer A to remove the free NPM, the NPM-actin was diluted to 0.4μM and treated by addition of increasing volume of $K_2PtCl_4$ solution. After standing overnight at 4°C, fluorescence intensity was measured with excitation wavelength at 342nm and emission wavelength at 375nm.

CD Measurements

CD spectra were recorded at room temperature on a Jasco-500c Polarizing Spectrophotometer. G-actin concentration was 0.4μM and the $K_2PtCl_4$ concentration varied in the range of 0-40μM. The spectra were scanned from 240nm to 190nm and repeated four times at sensitivity $2m°cm^{-1}$.

MSL-EPR Measurements

Labeling of G-actin with N-(1-oxyl-2,2,6,6-tetra-methyl-4-piperidinyl) maleimide was carried out as described by Galazkiewicz [12] with some modifications. G-actin was treated with maleimide (0.5mol/per mole of G-actin) at 4°C for 8h. KCl and $MgCl_2$ solutions were added to induce G-actin association and F-actin was then sedimented by ultracentrifugation. The pellet was homogenized and dialyzed against buffer A to remove the unbound dye. After dialysis, the G-actin solution was clarified by ultracentrifugation and concentrated to 5mg/ml. In 100μl concentrated G-actin solution, 0-9μl $K_2PtCl_4$ solution was added and various volume of buffer A was used to keep the final volume at 110μl of all samples. The samples were incubated at 22°C for 4h.

EPR measurements were performed at 22°C using a Bruker ESP-300 instrument. It was operated at scan range 10mT, radiation power 10mW, modulation amplitude 0.23mT, and field modulation 100KHz.

### Results

### Intrinsic Fluorescence

The titration curve based on intrinsic fluorescence measurement(Fig.1) shows that the fluorescence was quenched by $PtCl_4^{2-}$ continuously with the increasing of $PtCl_4^{2-}$ concentration.

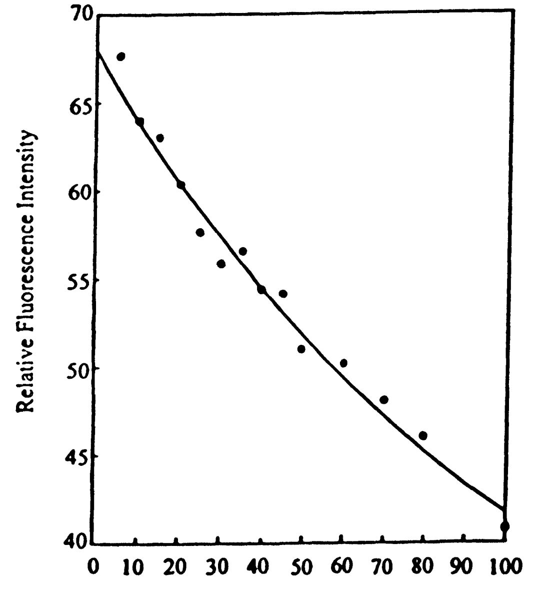

FIGURE 1. Intrinsic fluorescence of G-actin in presence of increasing $PtCl_4^{2-}$/actin molar ratio. $C_{actin}$=0.4μM, buffer A.

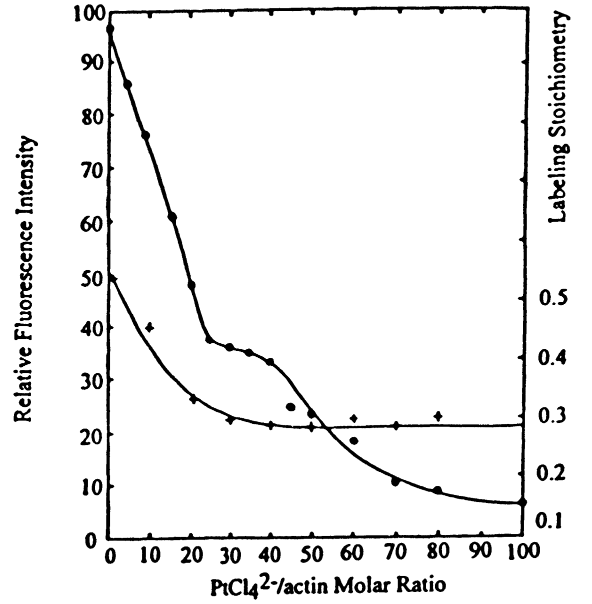

FIGURE 2. Curve A(•): the change in fluorescence of NPM labeled $PtCl_4^{2-}$ bound G-actin as a function of $PtCl_4^{2-}$/actin molar ratio.
Curve B(+): the effect of previous adding $PtCl_4^{2-}$ on the degree of labeling(mole NPM/mole actin). Experimental conditions were the same as described in the text. $C_{actin}$=0.4μM, buffer A.

### NPM Labeled Fluorescence

### The Reaction of $PtCl_4^{2-}$-Actin Complexes with NPM

It is known that NPM binds to cysteine-374 residue of G-actin specifically. NPM itself is nonfluorescent in aqueous solution but forms strongly fluorescent condensation product with sulfhydryl groups of organic compounds or proteins. If G-actin was treated previously with $K_2PtCl_4$ and then labeled with NPM, NPM reacted with the sulfhydryl of cys374 unoccupied by $PtCl_4^{2-}$. The dependence of fluorescence intensity on the concentration of $PtCl_4^{2-}$, as shown in Fig.2A., has a two-step profile. In the first step, a monotonous decrease in fluorescence intensity was found in the range of molar ratio $PtCl_4^{2-}$/actin(R)=0-25, and after then, a plateau appeared up to R=40. The data before the plateau were processed by Scatchard approach[13,14] as following.

From the linearity of this part, it is reasonable to assume that at low $PtCl_4^{2-}$concentration, the $PtCl_4^{2-}$ binding sites fall into one category and are mutually independent, the number (v) of $PtCl_4^{2-}$ bound to one G-actin molecule might be expressed as:

$$v=n_1k_1C_f/(1+k_1C_f) \qquad (1)$$

Here, $n_1$=number of $PtCl_4^{2-}$ binding sites in the first step, $k_1$= binding constant of these binding sites and $C_f$=concentrations of the unbounded $PtCl_4^{2-}$. C is the sum of the concentration of the bound and unbound ions:

$$C=C_f+C_b \qquad (2)$$

where $C_b$ refers to the concentration of the bound ions and depends on v and the analytical concentration of G-actin, $C_a$:

$$C_b=vC_a \qquad (3)$$

Combining the above equation with Eqns. 1 and 2, we obtain:

$$C=v/k_1/(n_1-v)+vC_a \qquad (4)$$

We define NPM fluorescence intensity(F) as the conformational change parameter, and assume that all bound ions contribute equally to the change in F. Consequently, there must exist a linear relation between v and F:

$$\Delta F=F_0-F=av \qquad (5)$$

The parameter, a, is a measure of the structure change in G-actin by the binding of a single $PtCl_4^{2-}$ to G-actin. $F_0$ is the fluorescence intensity at zero $PtCl_4^{2-}$ concentration. At very high $PtCl_4^{2-}$ concentrations, v becomes $v_{max}$ and is given by:

$$v_{max}=n_1=\Delta F_{max}/a \qquad (6)$$

Combining this expression with Eqn.4 and 5, we have

$$C=\Delta F/k_1/(\Delta F_{max}-\Delta F)+C_a\Delta F/a \qquad (7)$$

$\Delta F$ and $C_a$ are variables. A least-square fit of the experimental results gives $k_1=1.0x10^7M^{-1}$ with a=2.1 and $n_1$=30.

From the data in the second step, the binding constant $k_2$ and the number of binding sites $n_2$ were obtained as follows.

$C_b$ was supposed to be equal to the analytical $PtCl_4^{2-}$ concentration at the beginning of $PtCl_4^{2-}$ binding to those new sites. Fluorescence intensity (F) and $C_b$ can be expressed as:

$$C_b=(106-F)/4.55$$

With the $C_b$ values obtained from the equation, the $C_f$ and v were calculated. The best fit analysis showed that $PtCl_4^{2-}$ binding sites fall in one group in this step (linear regression coefficient r=-0.90) with $k_2=2.1x10^6M^{-1}$ and $n_2=26$.

With increasing $PtCl_4^{2-}$ concentration from 0 to 10μM (R=0-25), the molar ratio of the conjugated dye to actin was reduced significantly in NPM labeling $PtCl_4^{2-}$-actin complexes, which is reflected in the reduction of fluorescence intensity. When $C_{PtCl_4^{2-}}$ >10μM (R>25), the labeling stoichiometry is essentially unchanged(Fig.2B). The results reveal that Cys374 is involved in the first step(but not in the second step). The number of free thiols accessible to NPM decreased as the result of the occupation of a fraction of Cys374 thiol groups by previous platinum binding.

The Reaction of NPM Labeled G-actin with $PtCl_4^{2-}$

When labeled G-actin was titrated by $K_2PtCl_4$, the profile of the change in fluorescence intensity (Fig.3) resembles that given in Fig.2A, but the plateau appeared at lower R range (R=10-20).

The two-step binding data were analyzed separately by the methods mentioned above. The parameters obtained are as fllowing:

in first step, $k_1'=2.3x10^7M^{-1}$, a=2.5 and $n_1'=10$

in second step, $k_2'=3.2x10^6M^{-1}$, $n_2'=20$ (r=-0.92)

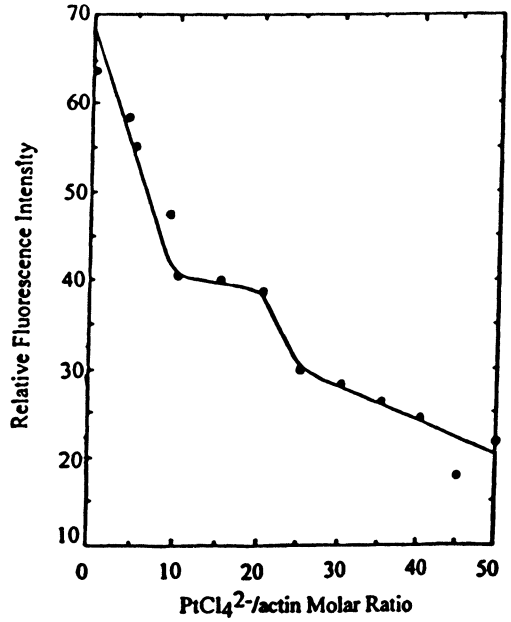

FIGURE 3. The effect of $PtCl_4^{2-}$ on NPM fluorescence of NPM-Actin. G-actin was previously labeled with NPM and free NPM was removed completely and then titrated with $PtCl_4^{2-}$. $C_{actin}$=0.4μM, buffer A.

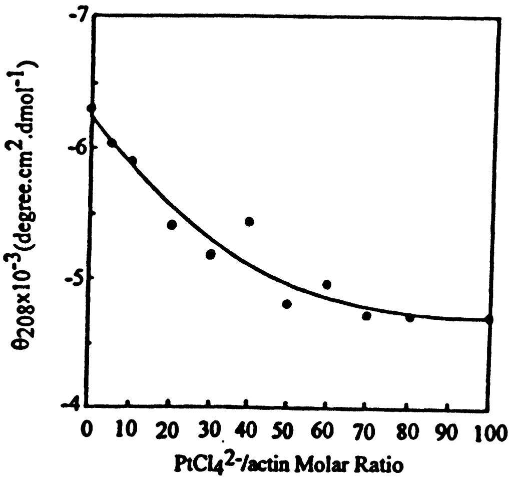

FIGURE 4. The dependence of $\theta_{208}$ of G-actin with $PtCl_4^{2-}$/actin molar ratio. $C_{actin}$=0.4μM, buffer A.

### CD Spectra

Fig.4 shows that the molar ellipticity $\theta_{208}$ is dependent on the concentration of $PtCl_4^{2-}$. When R is less than 50, $\theta_{208}$ values shift toward less negative values, indicating the changes of the secondary structure of G-actin. For a higher R, no further significant change in the secondary structure was observed.

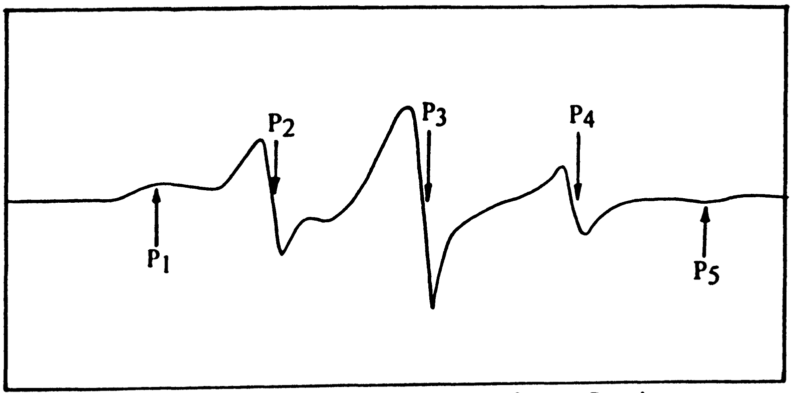

FIGURE 5. A typical EPR spectrum of MSL-G-actin. Experimental conditions were given in the text.

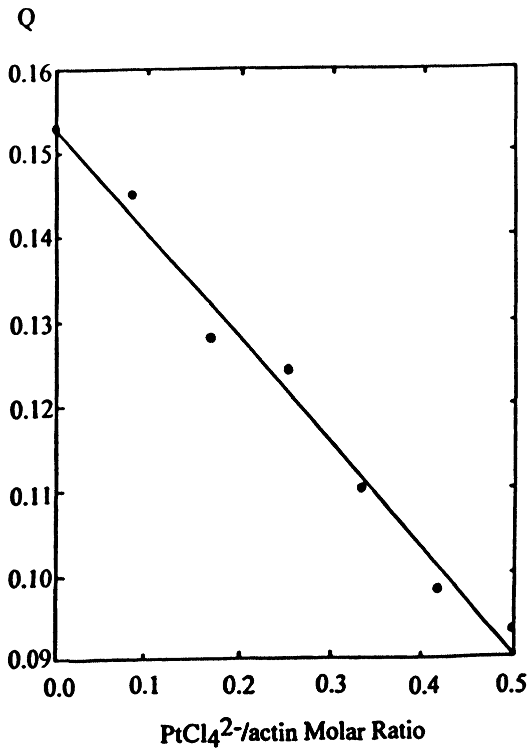

FIGURE 6. The effect of $PtCl_4^{2-}$ on Q value of MSL-G-actin.$C_{actin}$=0.4μM, buffer A. Experimental conditions were given in the text.

MSL-EPR Studies

Like many other proteins labeled with maleimide spin label, G-actin exhibits an EPR spectrum composed of two components, indicating an extremely immobile and a highly mobile fraction of the spin label. As shown in Fig.5, $P_2$ and $P_4$ are mainly due to very rapidly tumbling spins, whereas $P_1$ and $P_5$ are due to very slowly tumbling labels. In the middle of the spectrum ($P_3$) both components are superimposed. A change in the protein structure can be detected either by a change in the correlation times of the two components or by a change in the amplitude ratio of these components. We chose the ratio(Q) of the height of peaks $P_1$ and $P_2$, and the correlation time $\tau_c$ of rapidly tumbling spin labels to observe the effect of $PtCl_4^{2-}$on G-actin.

$\tau_c$ (in second)is obtained from the following equation[15]:

$\tau_c=6.5x10^{-10}\Delta H_o[(h(0)/h(-1))^{1/2}-1]$

here $\Delta Ho$ is the peak-to-peak distance of $P_3$, and h(0), h(-1) are the peak-to-peak heights of the $P_3$ and $P_4$, respectively.

Since MSL-EPR is a very sensitive way to get the information of conformational changes induced by the complexes such as $PtCl_4^{2-}$, it can be used to study interactions between $PtCl_4^{2-}$ and G-actin in R<1. As shown in Fig.6, Q decreased with increasing R. $\tau_c$ varied from $10.5x10^{-10}$s to $7.9x10^{-10}$s.

**Discussion**

The clear-cut two steps with a plateau in between, as shown in the fluorescence intensity-$PtCl_4^{2-}$ concentration curves(Fig.2A and 3) implicate that the interaction between $PtCl_4^{2-}$ and G-actin has a two-step feature. In the first step, $PtCl_4^{2-}$ binds to high-affinity sites, which can be characterized by $k_1=1.0x10^7M^{-1}$ and $n_1=30$. The platinum binding induces reduction in $\alpha$-helix content from the beginning and the exposure of tryptophane residue and the labeled Cys374 to hydrophilic environment. All these changes are related to molar ratio R. At this step, Cys374 residue is involved as deduced by comparing the curves in Fig.2A and 3. The previous occupation of Cys374 by NPM(in Fig.3) blocks $PtCl_4^{2-}$ binding and thus $n_1 < n_1$. Another evidence supporting Cys374 to be one of high-affinity groups is that the labeling molar ratio (NPM/actin) was reduced significantly with increasing $PtCl_4^{2-}$ concentration in this stage.The MSL-EPR enables us to get insight into the effect of $PtCl_4^{2-}$ binding in very low R value. The decreased Q value and $\tau_c$ which show that the freedom of thiols increased even in R below 1. It suggests the conformation of G-actin is becoming open from the initial stage of bound $PtCl_4^{2-}$.

The plateau in Fig.2A and 3 suggest that the non-specific binding in this stage did not make Cys374-related conformational change. The tryptophane residues is facing the more hydrophilic environment; nevertheless, the reduction in α-helix content becomes less pronounced. That means after the high-affinity sites accessible in the first stage are practically saturated, the C-terminal is little affected by further binding, but the whole molecular conformation becomes more opened continuously. The effect is accumulated and the deep-buried binding sites turn out to be accessible. Then the second step $PtCl_4^{2-}$ binding and conformational changes begin. Generally speaking, in this step, $PtCl_4^{2-}$ binds to sites of low-affinity ($k_2=2.1x10^6M^{-1}$, $n_2=26$). As the results show the NPM-labeled thiols move to hydrophilic environment again. While the quenching in intrinsic fluorescence goes on, no change in helix content was found.

In summary, with increasing in $PtCl_4^{2-}$ binding, the G-actin turns from a somewhat compact protein to a loose one in a discontinuous mode. With NPM-labeled Cys374 and tryptophane residue as the conformation markers, we can observe this two- stepness.

It is quite interested that the two-step process also has been found in $Cd^{2+}$(soft acid too)-spectrin system[17], but not in $Mg^{2+}$, $Ca^{2+}$, $Ce^{3+}$(hard acids)-actin systems[18,19]. The relation between the two-stepness and the hardness of metal ions is open to be testified.

For the $Cd^{2+}$-spectrin system, the behavior is different, although there is two-step feature too. $Cd^{2+}$ ions mainly bind to the non-thiol groups at the first step to lead to the exposure of sulfhydryl groups and further stronger binding occured. Thus we postulate the rigidity of proteins determines the behavior of conformational change. The relatively loose structure of actin facilitates the $PtCl_4^{2-}$ binding to sulfur-containing groups at the beginning of binding. However spectrin is a rod shape and compact protein, and it is difficult for metal ions to directly bind to deep-buried thiols, though they are of high affinity to soft acids.

**Acknowledgements:**

The present work is supported by the State Commission of Science and Technology, and National Natural Science Foundation of China. We thank Professor Jingfen Lu and Rongchang Li for their suggestions.

**References**

1. B. Rosenberg, Biochimie 60, 859(1978).
2. A. L. Pinto and S. J. Lippard, Biochim. Biophys. Acta 80, 167(1985).
3. P. Kopf-Maier and S. K. Muhlhausen, Chem.-Biol. Interactions 82, 295-316(1992).

4. Y.-X. Su et al., J. Mol. Sci.(China) 6, 105-110(1990).
5. S. K. Aggarwal and A. Sodhi, Cytobiology 7, 366(1973).
6. S. K. Aggarwal, J. Clin. Hematol. Oncol. 7, 760(1977).
7. J. D. Pardee and J. A. Spudich, Methods in Cell Biology 24, 271-288 (1982).
8. R. Cooke, Biochemistry 14, 3250-3256(1957).
9. U. K. Laemmli, Nature 227, 680-685(1970).
10. C. W. Wu, L. R. Yarbtough and J. Y. -H. Wu, Biochemistry 15(13), 2863-2868 (1976).
11. Y. Kawasaki, K. Mihashi, H. Janaka and H. Qhnuma, Biochim. Biophys. Acta 446,166-178(1976).
12. B. Galazkiewicz, J. Belagyi and R. Dabrowska, Eur. J. Biochem. 181, 607-614(1989).
13. B. Lammel and G. Maier, Biochim. Biophys. Acta 622, 245-258(1980).
14. C. Tanford, Physical Chemistry of Macromolecules, John Wiley, New York, 1961, pp. 526-586.
15. A. Keith, G. Bulfield and W. Snipes, Biophys. J. 10, 618(1970).
16. H.-H. Zeng, Thesis of Doctorate, Inorg. Chem. Dept. of Beijing Medical Univ.1991.
17. H.-Y. Sun, X.-L. Ma, T. Wang, R.-C. Li and K. Wang, Main Group Metal Chemistry 17, 665(1994).
18. M.-F. Carlier, D. Pantaloni and E. D. Korn, J. Biol. Chem. 261, 10778-10784(1986).
19. H. -Y. Sun, Q. Liu, Juan Zou and K. Wang, results unpublished.

**Received: October 24, 1994 - Accepted: November 7, 1994 - Received in revised camera-ready format: November 22, 1994**

